# Multilevel assessment of restaurant profitability: Evidence with European data

**DOI:** 10.1016/j.dib.2020.105426

**Published:** 2020-03-16

**Authors:** Miguel Díaz-Puche, Sergio M. Fernández-Miguélez, Juan A. Campos-Soria, Manuel A. Fernández-Gámez

**Affiliations:** a University of Málaga, Campus Teatinos 29071, Málaga, Spain; b Department of Applied Economics, University of Málaga, Campus El Ejido 29071, Málaga, Spain; c Department of Finance and Accounting, University of Málaga, Campus El Ejido 29071, Málaga, Spain

**Keywords:** Restaurant profitability, Multilevel analysis, Tourism industry, Financial performance, European data

## Abstract

Previous literature has analysed the effects of establishment regressors on different measures of restaurant financial performance [1,2]. However, as [3] stated, these studies have focused on the analysis at establishment level rather than at corporate level. Determining the factors that explain the restaurant profitability is not only an important phenomenon for establishments but also for companies because they adapt their products, services and strategies to obtain additional benefits and cash flow [1]. Additionally, progressive globalization has forced companies to operate in countries with environments that differ from the companies’ country of origin [4]. In this context, the dataset presented in this paper sought to contribute to the existing literature in two ways. First, it allows us to investigate the factors that determine the profitability of restaurant corporations using advanced measures of financial performance. Second, a multilevel experimental design may be helpful when understanding country heterogeneity in companies´ profitability. The dataset contains a sample of 860 restaurant corporations operating in 18 European countries. From each corporation in the sample 6 financial variables were collected, and from each country, 10 context variables associated with economic conditions and tourism environment were considered. Due to the lack of data that allow a global analysis of the factors that determine profitability in the restaurant industry, this dataset can play an important role for business management, which should control not only their financial ratios but also the macroeconomic conditions and tourism environment where the companies operate.

Specifications table

**Table:** 

Subject	Economics and Econometrics
Specific subject area	Tourism and Hospitality Management
Type of data	Table, Figure
How data were acquired	Microeconomic data provided by Amadeus database, and contextual variables provided by World Tourism Organization, OECD, and Eurostat
Data format	Raw, analysed, descriptive and inferential statistical data
Parameters for data collection	Two measures of corporate profitability are considered in this dataset: Return on equity (ROE) and Return of assets (ROA). Additional financial indicators are included as control variables. Country heterogeneity is checked using macroeconomic variables and tourism indicators. Within and between-country variability may be decomposed using a hierarchical data experiment.
Description of data collection	Data were collected by using a multi-step approach. First, we use a random sampling technique for financial indicators. Second, we consider different international data set for contextual variables
Data source location	Countries included: Austria, Belgium, Denmark, Finland, France, Germany, Hungary, Ireland, Italy, Latvia, Lithuania, Norway, Poland, Portugal, Romania, Spain, Sweden, and United Kingdom
Data accessibility	Data is included in this article. Also publicly available at repository Mendeley: https://data.mendeley.com/datasets/b8mc5byy7g/1

## Value of the data

• This data set can be beneficial to analyze the factors that determine the profitability of corporations in the restaurant industry.
• This dataset can be used to conduct different studies on the heterogeneous pattern that profitability of European restaurant corporations presents at country level. These studies would benefit from the creation of company profiles within each country based on financial determinants of profitability.
• The data can be useful for business managers and public institutions with an interest in tourism. Understanding the causes of heterogeneity among countries provides useful insights for companies´ business growth and location strategies.
• The analysis is useful when discovering new and innovative sources of strategic competitive advantages.

## 1 Data

Data were collected by using a multi-step approach to address their objectives with solvency. The first dataset was obtained through a sample consisting of 860 restaurant corporations selected from the total population of active restaurants in Europe in 2018. A random sampling technique was applied to stratify 18 European countries with a sample error of less than 1%. This dataset includes financial indicators obtained from the corporate financial statements from Amadeus Database by Bureau Van Dijk. The second dataset concerned contextual variables and shows macroeconomic and tourism indicators related to the countries in which the restaurant corporations in the sample are active.

The data file spreadsheet accompanying this article consists of 860 rows and 20 columns of data. The first four columns collect the identification code of the company and the country where the company operates. The rest of the columns include financial indicators and contextual variables. In the group of financial indicators, two measures of corporate profitability are considered: Return on equity (ROE) is calculated as profits available to equity shareholders/equity shareholders’ funds of corporate and Return of assets (ROA) is calculated as earnings before interest and tax/total assets. In addition, information is also included on other financial indicators such as Fixed assets ratio (net sales/average net fixed assets) [2], Current ratio (current assets/current liabilities) [5], Log total assets (natural logarithm of the total assets) [2], and Debt equity ratio (total liabilities/shareholders’ equity) [6]. Likewise, the macroeconomic variables in the data file spreadsheet are the following: per capita GDP in euros (gross domestic product current prices, euros per capita), per capita GDP in PPS (gross domestic product current prices, purchasing power standard per capita), GDP growth (real GDP growth rate, gross domestic product at market prices), Interest rate (short-term interest rates,%), Inflation rate (annual average rate of change,%, Harmonised Index of Consumer Prices), and Public debt over the GDP (general government gross debt - quarterly data, percentage of gross domestic product). All these macroeconomic indicators have been collected from Eurostat, excepted the interest rate, which was obtained from OECD. Additionally, we add different inbound tourism indicators obtained from UN World Tourism Organization and Eurostat: Tourism intensity (nights spent at tourist accommodation establishments per thousand inhabitants), ITE over GDP (inbound tourism expenditure over GDP,%), Trips (number of trips by country of destination), and Nights (number of nights spent by country of destination). Table 1 shows the descriptive statistics for the complete sample. The mean of ROE is 18.94% and the mean of ROA is 5.92%. This difference indicates a high level of leverage in the industry, corroborated with the high value of Debt Equity Ratio (3.13).

**Table 1:** Descriptive statistics.

	Obs.	Mean	S.D.	Min.	Max.
**Microeconomic variables**					
ROE	860	0.1894	0.9724	−10.9906	5.8409
ROA	860	0.0592	0.1441	−0.5243	0.9742
Fixed assets ratio	860	2.9323	2.6884	0.0099	23.1444
Current ratio	860	0.8218	2.9767	0.0000	48.4615
Log total assets	860	4.1030	0.5451	2.6570	5.7889
Debt equity ratio	860	3.1313	10.9857	−42.6934	97.1538
**Contextual variables**					
GDP pc (eur)	860	34,448.02	12,438.94	9580.00	66,950.00
GDP pc (PPS)	860	31,384.55	6000.463	18,800.00	54,850.00
GDP growth	860	2.5903	1.2371	1.70	8.10
Interest rate	860	0.1158	0.6185	−0.695	2.25
Inflation rate	860	2.0219	0.6901	0.30	3.70
Public debt over GDP	860	83.8411	27.4141	36.00	136.40
Tourism intensity	860	5690.334	2304.495	1370.14	13,806.95
ITE over GDP	860	2.7687	1.6643	1.4186	9.5969
Trips	860	7.20e+07	6.79e+07	3,888,795	2.21e+08
Nights	860	3.53e+08	2.84e+08	1.08e+07	9.70e+08

**Fig. 1:**
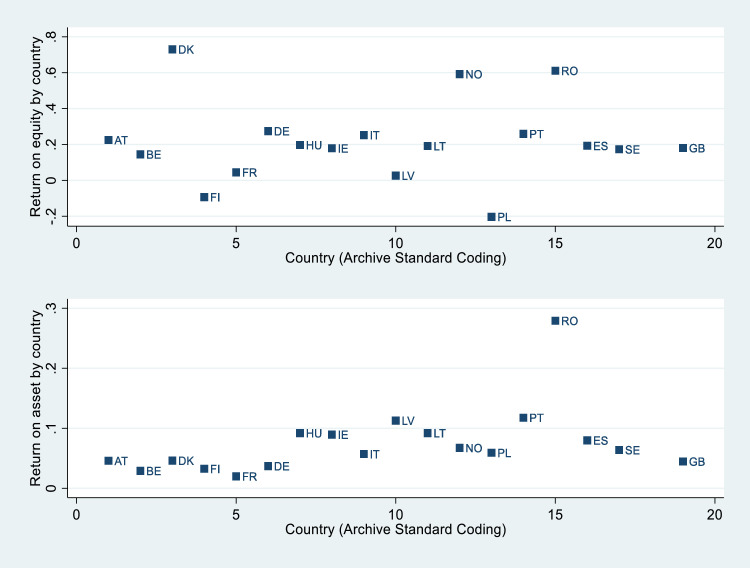
ROE and ROA by country.

Fig. 1 points out ROE and ROA by country, indicating that a country heterogeneity exits among corporations, stronger in relation to ROE. This information may indicate that it is necessary to investigate how certain contextual variables can influence the profitability levels of the sample corporations.

## 2 Experimental design, materials, and methods

**Fig. 2:**
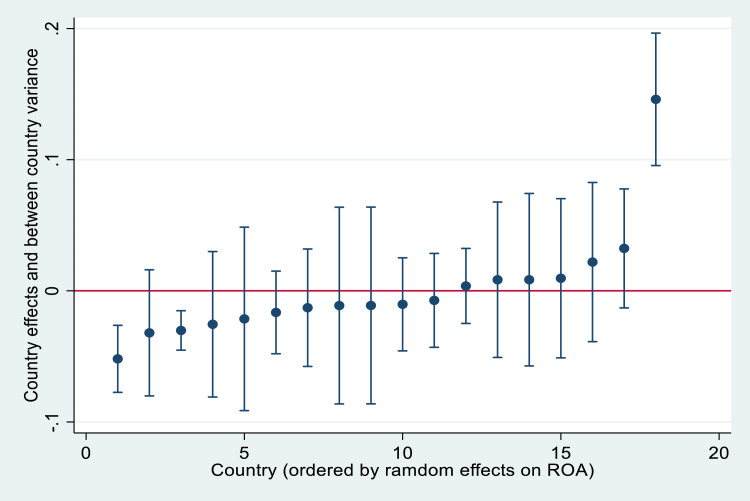
Country effect on ROA in rank order.

**Fig. 3:**
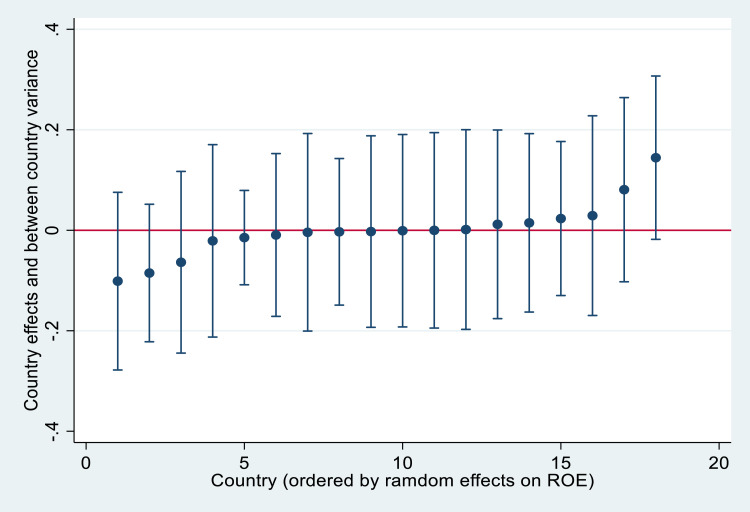
Country effect on ROE in rank order.

In general, the transnational analysis of the financial performance of restaurant corporations exhibits a hierarchical data structure, which does not match the statistical properties of a random sample. Specifically, companies from the same country share common economic and tourism environment conditions. Some of these characteristics are included in the dataset. However, other contextual variables may be unobservable and, although the coefficients estimated by standard regression remain consistent, standard errors may be biased [7]. Therefore, the proposed dataset allows us a multilevel design with hierarchical linear modeling, which simultaneously integrates both a microeconomic approach using companies’ financial predictors, and a macroeconomic approach in order to analyze national heterogeneity patterns [8]. In the two-level structure considered, companies (i.e. first level) are nested in countries (i.e. second level). We first apply a null model with an intercept and country effect, but without explanatory variables. This model is analogous to a random-effects ANOVA. Estimates from this model indicate strong evidence of statistically significant heterogeneity between countries, especially in ROA. Such heterogeneity is shown in Fig. 2, Fig. 3, in which the country effects and the between-country residual variance on ROA and ROE are presented, respectively.

**Fig. 4:**
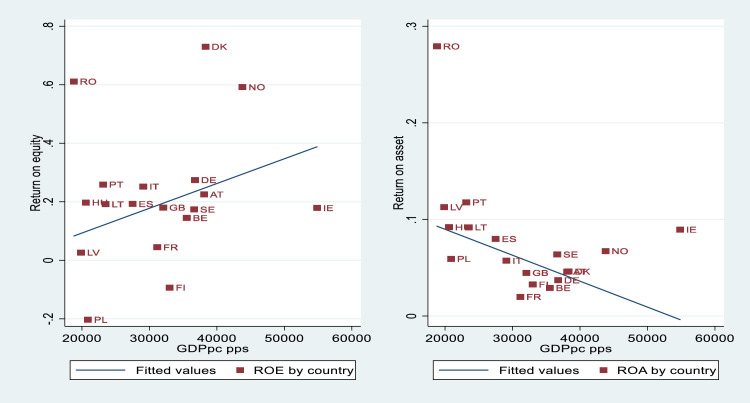
A cross-country analysis of profitability measures and GDPpc.

**Fig. 5:**
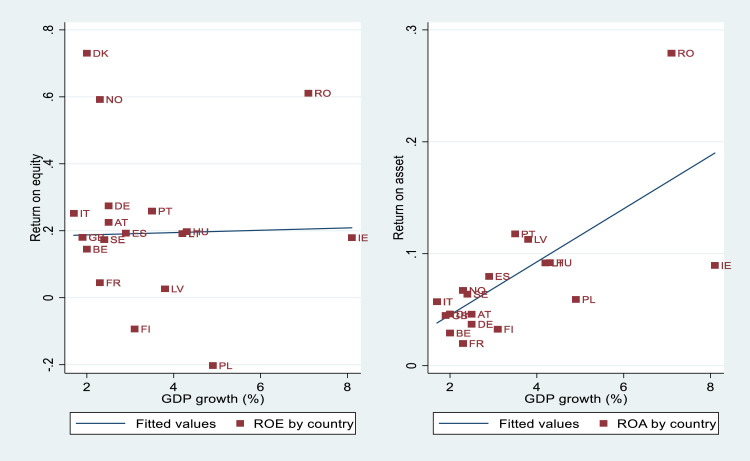
A cross-country analysis of profitability measures and GDP growth rate.

The figures above demonstrate that country effects vary randomly but also that significant variance exists in the level of financial performance within and between countries. We can explain the causes of such heterogeneity between countries, adding contextual regressors, which could help to understand the impacts of economic conditions and the tourism environment on corporate profitability. For instance, Fig. 4, Fig. 5 demonstrate the importance of per capita GDP and GDP growth when explaining country heterogeneity. These indicators allow controlling the purchasing power of residents, as well as the important of domestic tourism on financial performance. Additional macroeconomic indicators, such as interest rates, inflation rates, or public debt could also be relevant. The importance of inbound tourism should be also controlled by different tourism intensity indicators, which could explain, to a large extent, the existing variability among countries.

Therefore, the dataset associated to this paper sheds light on an important issue still inadequately explored in the literature on companies’ financial performance. Although traditionally, stakeholders have managed companies’ financial performance based on their economic and financial indicators, the dataset shows additional indicators related to the country in which a company operates, which allows us to understand the causes of heterogeneity among countries in a context of internationalised companies and globalised markets. Future research using hierarchical linear models could facilitate the creation of company profiles based on both their financial variables and each country's specific economic and tourism environment. Moreover, the data included in this paper is also relevant to guide further research to improve knowledge of the determinants of corporates’ profitability in the restaurant industry.

## Conflict of Interest

The authors declare that they have no known competing financial interests or personal relationships that could have influenced the work reported in this paper.
